# Exposure to Concentrated Coarse Air Pollution Particles Causes Mild Cardiopulmonary Effects in Healthy Young Adults

**DOI:** 10.1289/ehp0900558

**Published:** 2009-03-23

**Authors:** Donald W. Graff, Wayne E. Cascio, Ana Rappold, Haibo Zhou, Yuh-Chin T. Huang, Robert B. Devlin

**Affiliations:** 1 MDS Pharma Services, Lincoln, Nebraska, USA; 2 Department of Cardiovascular Sciences, East Carolina University Brody School of Medicine, Greenville, North Carolina, USA; 3 Human Studies Division, National Health and Environmental Effects Research Laboratory, U.S. Environmental Protection Agency, Research Triangle Park, North Carolina, USA; 4 School of Public Health, University of North Carolina at Chapel Hill, Chapel Hill, North Carolina, USA; 5 School of Medicine, Duke University, Durham, North Carolina, USA

**Keywords:** cardiovascular effects, coarse PM human study

## Abstract

**Background:**

There is ample epidemiologic and toxicologic evidence that exposure to fine particulate matter (PM) air pollution [aerodynamic diameter ≤ 2.5 μm (PM_2.5_)], which derives primarily from combustion processes, can result in increased mortality and morbidity. There is less certainty as to the contribution of coarse PM (PM_2.5–10_), which derives from crustal materials and from mechanical processes, to mortality and morbidity.

**Objective:**

To determine whether coarse PM causes cardiopulmonary effects, we exposed 14 healthy young volunteers to coarse concentrated ambient particles (CAPs) and filtered air. Coarse PM concentration averaged 89.0 μg/m^3^ (range, 23.7–159.6 μg/m^3^). Volunteers were exposed to coarse CAPs and filtered air for 2 hr while they underwent intermittent exercise in a single-blind, crossover study. We measured pulmonary, cardiac, and hematologic end points before exposure, immediately after exposure, and again 20 hr after exposure.

**Results:**

Compared with filtered air exposure, coarse CAP exposure produced a small increase in polymorphonuclear neutrophils in the bronchoalveolar lavage fluid 20 hr postexposure, indicating mild pulmonary inflammation. We observed no changes in pulmonary function. Blood tissue plasminogen activator, which is involved in fibrinolysis, was decreased 20 hr after exposure. The standard deviation of normal-to-normal intervals (SDNN), a measure of overall heart rate variability, also decreased 20 hr after exposure to CAPs.

**Conclusions:**

Coarse CAP exposure produces a mild physiologic response in healthy young volunteers approximately 20 hr postexposure. These changes are similar in scope and magnitude to changes we and others have previously reported for volunteers exposed to fine CAPs, suggesting that both size fractions are comparable at inducing cardiopulmonary changes in acute exposure settings.

The U.S. Environmental Protection Agency (EPA) currently regulates particulate matter (PM) on the basis of mass in two size ranges: coarse PM [2.5–10 μm in aerodynamic diameter (PM_2.5–10_)] and fine PM (PM_2.5_). Coarse PM typically derives from soil or abrasive mechanical processes in transportation or industry, and also can contain biogenic materials such as pollen, endotoxin, and mold spores known to have deleterious effects on human health, especially in those with pulmonary diseases such as asthma. In the western United States, coarse PM typically comprises > 50% of measured PM_10_ mass on an annual basis, and approaches 70% in some areas of the Southwest. Fine PM derives primarily from combustion processes and may contain both primary particles directly emitted from specific sources and secondary particles formed by atmospheric chemistry changes over time. Wilson and Suh (1997) provide a detailed description of the occurrence and composition of coarse and fine PM and conclude that they are separate classes of pollutants and should be measured separately in epidemiology and toxicology studies.

In addition to difference in chemical composition, these two size fractions deposit in different locations in the lung, with coarse PM predominantly depositing in the more proximal portion of the lung (Kim and Hu 1998). Because lung deposition and chemical composition of fine and coarse PM are generally dissimilar, PM may produce biologic activity and health effects distinct in nature and severity from those effects seen with exposure to fine PM.

Epidemiology studies using time-series analyses have demonstrated significant correlations between exposure to ambient PM air pollution and increased mortality and morbidity. Although most time-series studies have demonstrated health effects to be more strongly correlated with the PM_2.5_ fraction than with the PM_2.5–10_ fraction (Cifuentes et al. 2000; Klemm et al. 2000; Lipfert et al. 2000; Schwartz and Neas 2000), there is some evidence for effects of coarse PM on mortality and morbidity, especially in arid regions where coarse PM concentrations are relatively high (Castillejos et al. 2000; Mar et al. 2000; Ostro et al. 2000). Epidemiologic evidence of health effects associated with coarse PM was recently reviewed (Brunekreef and Forsberg 2005).

A panel study of 19 nonsmoking older adults with cardiovascular disease residing in the Coachella Valley, California, reported an association between decreased heart rate variability (HRV) and coarse PM (Lipsett et al. 2006). A panel study of 12 adult asthmatics residing in Chapel Hill, North Carolina, reported associations between HRV, blood lipids, and circulating eosinophils and coarse, but not fine, PM (Yeatts et al. 2007).

Recently, virtual impactor technology (Demokritou et al. 2002; Kim et al. 2000) has been used to expose humans to concentrated fine ambient PM. These studies have reported that fine concentrated ambient particles (CAPs) can cause mild pulmonary inflammation and increased blood fibrinogen in healthy volunteers (Ghio et al. 2000, 2003), decreased HRV in healthy elderly volunteers (Devlin et al. 2002), decreased arterial oxygenation and HRV in elderly volunteers who are healthy but have chronic obstructive pulmonary disease (Gong et al. 2004a), and increased HRV and mediators of blood coagulation in healthy and asthmatic subjects (Gong et al. 2003a). Additionally, exposure of healthy volunteers to fine CAPs plus ozone caused brachial artery vasoconstriction (Brook et al. 2002) and increased diastolic blood pressure (Urch et al. 2005), and these findings were attributed primarily to CAP constituents (Urch et al. 2004).

To understand more fully the pathologic effects of the coarse fraction and to determine biological plausibility of epidemiologic studies, we examined the physiologic effects of concentrated Chapel Hill coarse CAPs on several cardiac, blood, and pulmonary end points in young, healthy volunteers. This study is part of a continuing series of studies in which humans are exposed to different size fractions of Chapel Hill PM and will also allow us to compare the relative toxicity of coarse and fine PM from the same geographic location. We presented a brief comparison of humans exposed to three size fractions of Chapel Hill CAPs in condensed form in a symposium proceedings (Samet et al. 2007).

## Materials and Methods

### Study population

This was a single-blind, crossover study approved by the Committee on the Protection of the Rights of Human Subjects of the University of North Carolina at Chapel Hill School of Medicine. Before inclusion, participants were informed of the study procedures and risks, signed a statement of informed consent and were approved for participation by a U.S. EPA physician. Screening procedures included medical history, physical exam, and routine hematologic and biochemical tests. Specific exclusion criteria included any chronic medical condition or chronic medication use (except birth control pills, low-dose antibiotics for acne, or dietary supplements), significant risk factors for cardiovascular disease (e.g., high cholesterol or uncontrolled blood pressure), and current smokers or those with a significant smoking history within 1 year of study participation. Subjects suffering from seasonal allergies were not studied within 6 weeks of a symptomatic episode, and no subject was studied within 4 weeks of a respiratory tract infection. Individuals unable to discontinue substances that could potentially alter their inflammatory response (e.g., antioxidants, nonsteroidal anti-inflammatory agents) for at least 6 days before exposure were not allowed to participate. A urine pregnancy test was performed on all females during the screening process and before each exposure.

### Exposure of subjects to coarse CAPs

Ambient air was drawn into an inlet duct on the roof of the U.S. EPA human studies facility (~ 30 m above ground level) in Chapel Hill at 5,000 L/min and transferred downward into the building to a PM_10_ inlet of the concentrator. The size selective inlet and the virtual impactors used to concentrate particles are identical to those developed by Demokritou et al. (2002). The major outward flow was 4,500 L/min for the first impaction stage and 450 L/min for the second impaction stage, giving a 50 L/min concentrated aerosol outflow. We diluted the concentrated aerosol with 150 L/min of room-temperature dilution air [relative humidity (RH) ~ 30%], which was added to provide enough airflow for the human subjects’ breathing requirements. The dilution air was passed through HEPA filters and charcoal to strip it of particles and gaseous air pollutants. We assessed PM concentrations in the chamber by gravimetric analysis of filters. We placed the filters immediately upstream of the inlet duct to the chamber, about 30 in from the subject’s mouth. We measured real-time concentrations upstream of the concentrator and in the chamber using a Fisher Scientific DataRam 4 monitor (Franklin, MA) and used these concentrations to calculate the concentration factor, which ranged from 4- to 10-fold. We measured particle size distributions with a 3321 APS instrument (TSI Inc., St Paul, MN). Because coarse PM is not evenly distributed in a chamber of this size, each subject was seated with their mouths about 12 in from the chamber inlet duct. Even at that close distance, the subjects inhaled only 67% of the particles measured at the filter inlet. Table 1 shows the actual dose of particles inhaled by each subject.

Exposures were conducted in a specially designed Plexiglas chamber at approximately 20°C and 40% RH. Participants were randomly exposed to filtered air and concentrated PM_2.5–10_ for 120 min, with intermittent exercise, on two separate occasions separated by at least 1 month. The exercise schedule consisted of 15 min of rest followed by 15 min of exercise on a recumbent bicycle, repeated four times over the 120-min exposure session, with a target ventilation of 20 L/min/m^2^ during exercise. During a training session, the workload needed for each subject to achieve the target minute ventilation was calculated. Most subjects achieved the target using a work load of 75 W; however, the tension on the exercise bike was adjusted according to each subject’s calculations.

### End point measurements

#### Bronchoscopy with lavage

Subjects underwent bronchoscopy with lavage approximately 20 hr after the completion of each exposure, as described in detail in Huang et al. (2006). We quantified cell numbers by counting in a hemocytometer and assessed viability by exclusion of trypan blue dye. Viability exceeded 90% in all samples. Cells were stained using DiffQuik reagents purchased from Sigma (St. Louis, MO), and we determined differential cell counts [polymorphonuclear neutrophils (PMNs), macrophages, lymphocytes, monocytes, epithelial cells, and eosinophils] by counting at least 300 cells under an oil-emersion lens. We stored bronchial lavage (BL) and bronchoalveolar lavage (BAL) fluid at −80°C, and at the end of the study we used commercially available kits to quantify levels of interleukin (IL)-6 and IL-8 (R&D Systems, Minneapolis, MN), prostaglandin E_2_ (PGE_2_; New England Nuclear, Boston, MA), α1-antitrypsin (ALPCO Diagnostics, Windham, NH), and total protein (BioRad, Hercules, CA).

#### Spirometry

We assessed pulmonary function with a SensorMedics Vmax system (VIASYS, Conshohocken, PA). We took measurements of forced vital capacity (FVC), forced expiratory volume in 1 sec (FEV_1_), and carbon monoxide diffusion capacity (DLCO) before, after, and approximately 20 hr after the completion of each exposure as described earlier (Ghio et al. 2000).

#### Cellular and soluble blood components

We collected venous blood immediately before and approximately 1 hr and 20 hr after the completion of each exposure. Basic blood chemistry, complete blood count with differential, and serum catecholamines were measured by LabCorp (Burlington, NC) on the day the blood was drawn. Plasma and serum was also frozen at −80°C for later analysis. We used commercially available ELISA kits to quantify levels of C-reactive protein (ALPCO Diagnostics, Windham, NH); D-dimer and von Willebrand’s factor (vWF; Diagnostica Stago, Parsippany, NY); factor VII, factor IX, prothrombin, tissue plasminogen activator (tPA), plasminogen, fibrinogen, and protein C (Enzyme Research Laboratories, South Bend, IN); and plasminogen activator inhibitor 1 (DakoCytomation, Carpenteria, CA).

#### Heart rate variability (HRV)

We collected continuous ambulatory electrocardiograms (ECGs) for approximately 24 hr using a 3100A Zymed Holter System (Phillips, Andover, MA) and processed them by standard Zymed algorithms. We sampled the ECGs at 120 Hz and stored all data on flash cards before processing. An electrocardiographic research nurse blinded to the particle exposure randomization then edited the sequence of ECG complexes to ensure proper labeling of each QRS complex. From the edited records, we assessed time domain [standard deviation of normal-to-normal intervals (SDNN) and percentage of differences between adjacent normal-to-normal intervals that are > 50 msec (PNN_50_)] and frequency domain [total, low-frequency (LF; 0.03–0.15 Hz), and high-frequency (HF; 0.15–0.4 Hz) power] variables for the 5-min time periods immediately before exposure and approximately 1 hr and 20 hr after the completion of each exposure. We conducted measurements while subjects reclined quietly in a dark room for 30 min, with the final 10 min being used for HRV analysis. In addition to the 5-min intervals, we also calculated SDNN and average SDNN for the entire 24-hr ambulatory monitoring period.

### Statistical analysis

We assessed lung function, blood, and Holter measurements immediately before, 1 hr after, and 20 hr after each exposure. We assessed BAL/BL measurements only 20 hr postexposure. For analysis, we subtracted preexposure values from 1 hr and 20 hr postexposure values and compared the air exposure differences with the CAP exposure differences for each person. Data in the figures are percent change in these differences per 10-mg/m^3^ increase in PM concentration in the exposure chamber. Because we took BAL measurements only 20 hr postexposure, we compared only those values during the statistical analysis. Concentration of coarse PM in the chamber is measured on a continuous scale and varies from subject to subject depending on the outdoor PM concentration that day. Table 1 shows the inhaled and chamber concentrations during the CAP exposure. PM concentrations in the chamber during air exposures were low, but not zero. We used linear mixed effects models (R statistical software, version 2.3.1, lmer package) to test differences in response between the CAP and filtered air exposures. More specifically, we used a random intercept model to account for the subject-level variability and estimated the slope parameter that described an expected change in response as a function of PM concentration. We summarize the estimates of slope on the basis of a 10-mg/m^3^ increase in PM concentration. We also report the mean and standard error associated with each end point. We used an α of 0.05 to determine statistical significance.

## Results

### Study population and exposure

**Figure 1:**
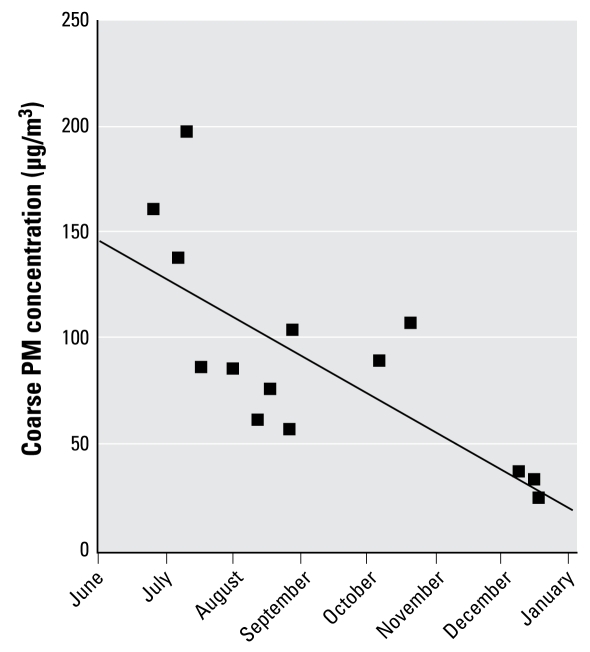
Correlation between coarse PM concentration in the exposure chamber and date of PM exposure. The solid line represents the linear regression line. The correlation of determination (*r*^2^) is 0.55 (*p* = 0.0025).

Table 1 contains basic demographic PM concentration information. This study enrolled six female and eight male participants with a mean age of 24.9 years. All exposures began within 30 min of 0945 hours to control for diurnal variations in physiologic response. Although we constructed the concentrator to concentrate only PM_2.5–10_, there is some concentration of PM_1–2.5_ (particles between 1 and 2.5 μm). “Total PM” in Table 1 refers to the combination of all particles present in the chamber. We observed substantial variation in concentrated PM exposure reflecting the natural daily variation in PM concentration outside the facility. CAP exposures took place from June to December, and, as expected, coarse PM concentrations were higher in the warmer months (Figure 1).

### Coarse PM caused small but significant changes in lung neutrophils and monocytes

**Figure 2:**
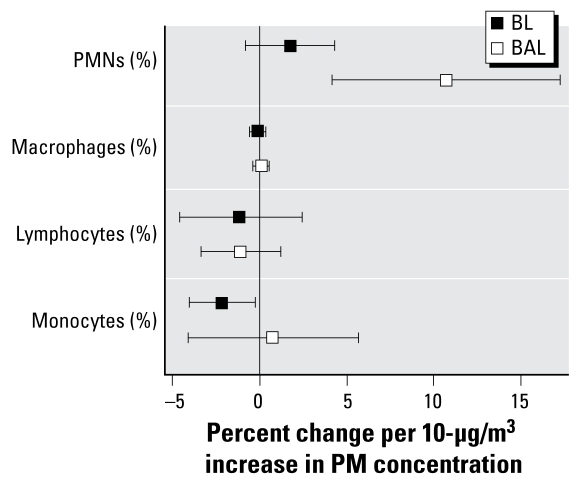
Changes in the percentage of each cell type in the BL and BAL fluids. Most participants had zero basophils and eosinophils in either fraction (data not shown).

Total cell recoveries in the BL and BAL fluids did not differ between air- and CAP-exposed individuals. Figure 2 provides data for the cell differential end points. In the BAL fraction we observed a statistically significant 10.7% increase in percent PMNs per 10 μg/m^3^ of coarse PM (*p* = 0.0065), indicative of mild pulmonary inflammation. BAL PMN values in air-exposed individuals were 1.0 ± 0.30%. In the BL fraction, we observed a trend toward an increase in PMNs with increasing CAP concentration, although the trend was not statistically significant. We observed a small, but statistically significant, 2.0% decrease in percent monocytes in the BL fraction (*p* = 0.05) per 10 μg/m^3^ of coarse PM. Monocyte levels in air-exposed individuals were 7.2 ± 0.8%. We observed no significant changes in any of the other cell types in either fraction.

### Coarse PM caused a decrease in total protein in the BAL

**Figure 3:**
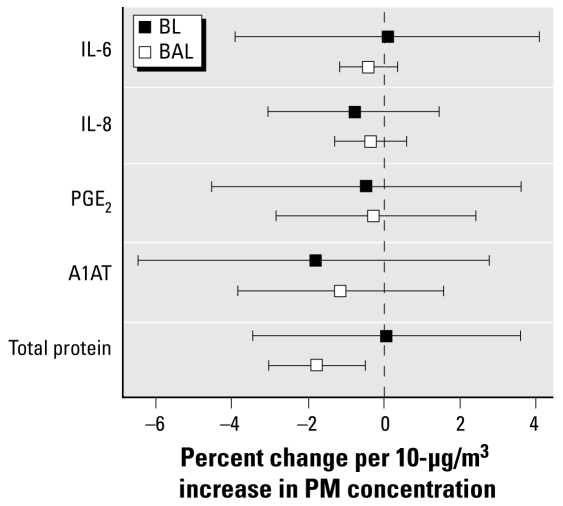
Changes in inflammatory mediators in the BL and BAL fluids. A1AT, α1-antitrypsin.

We observed no differences in recovery of BL or BAL fluid between air- and CAP-exposed individuals. The group average return was within 10%, so soluble components were expressed per milliliter of lavage fluid. As shown in Figure 3, soluble markers of inflammation present in BAL and BL fluids (IL-6, IL-8, PGE_2_) were not changed after exposure to coarse CAPs for 2 hr. However, we observed a 1.8% decrease in total protein in BAL fluid (*p* = 0.0191) per 10 μg/m^3^ of coarse PM. Protein levels in BAL fluid of air-exposed individuals were 74.2 ± 9.0 μg/mL. In an earlier study (Ghio et al. 2000), we also reported decreased protein in lavage fluid of humans exposed to fine CAPs.

### Coarse PM caused no changes in pulmonary function

**Figure 4:**
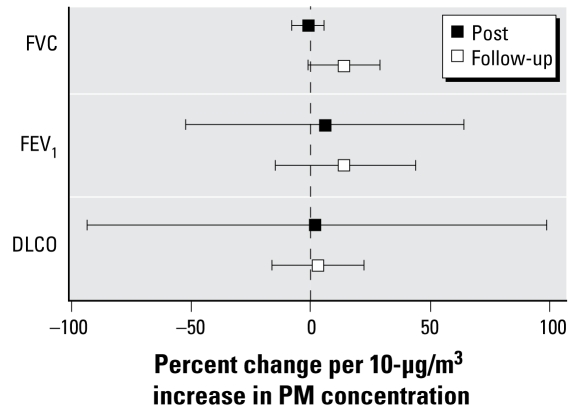
Changes in measures of pulmonary function: immediately after exposure (Post) and approximately 20 hr after exposure (Follow-up).

We took lung function measurements before, immediately after, and again 20 hr after exposure to air and coarse CAPs. Relative to measurements taken before exposure, FEV_1_ and FVC did not show statistically significant changes either immediately or 20 hr after exposure to coarse CAPs (Figure 4). A recent study reported changes in pulmonary diffusing capacity (DLCO) for carbon monoxide in humans exposed to ultrafine carbon black particles (Pietropaoli et al. 2004). However, in this study we saw no change in DLCO either immediately or 20 hr after CAP exposure.

### Coarse CAPs cause small changes in vascular factors involved in coagulation

**Figure 5:**
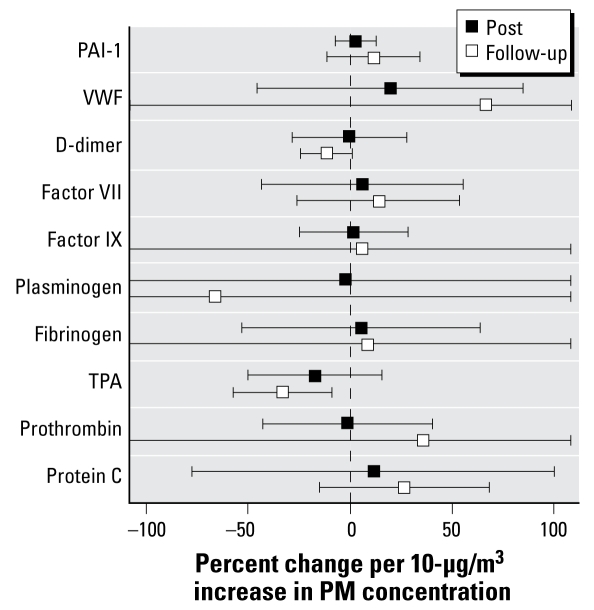
Changes in mediators of coagulation: immediately after exposure (Post) and approximately 20 hr after exposure (Follow-up). PAI-1, plasminogen activator inhibitor-1.

We measured a number of soluble factors involved in clotting and coagulation before, immediately after, and again 20 hr after exposure to air or coarse CAPs. Relative to measurements taken before exposure, only tPA showed a significant change after CAP exposure (Figure 5). tPA concentration decreased 32.9% from the mean baseline level (*p* = 0.01) per 10-μg/m^3^ increase in CAP concentration when measured approximately 20 hr after exposure. Decreased tPA levels could potentially result in the formation of less plasmin, a compound that plays a key role in dissolving blood clots that may have formed in blood vessels. D-dimer concentration decreased 11.3% per 10 μg/m^3^ of coarse PM 20 hr after exposure, but the decrease did not quite achieve statistical significance (*p* = 0.07). tPA and D-dimer concentrations in blood drawn before exposure were 6.5 ± 1.6 ng/mL and 349 ± 61 ng/mL, respectively.

### Other blood biomarkers

We also examined markers of systemic inflammatory processes, catecholamines, and lipids in the blood (data not shown). Coarse CAP exposure did not cause significant changes in C-reactive protein, catecholamines, triglycerides, or cholesterol (total, very-low-density, low-density, and high-density lipoproteins).

### HRV

**Figure 6:**
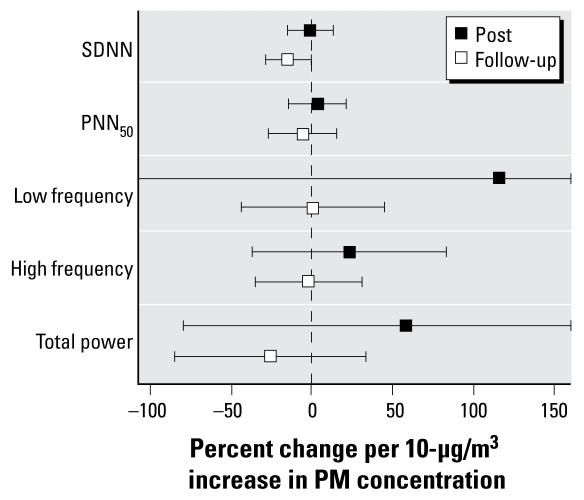
Changes in resting HRV measurements: immediately after exposure (Post) and approximately 20 hr after exposure (Follow-up).

In a previous study (Devlin et al. 2003), we reported that elderly healthy people exposed to fine CAPs had changes in both time- and frequency-domain variables as measured by Holter recording. In this study, we applied a Holter monitor to each subject before exposure, which the subject wore for 24 hr. We measured SDNN, a marker of overall HRV, using data from the entire monitoring period. We measured PNN_50_, LF, HF, and total power during three 5-min periods when the subject was lying at rest: immediately before, immediately after, and 20 hr after exposure to air or coarse CAPs. Relative to the measurements taken before exposure, SDNN decreased 14.4% from the mean baseline level per 10-μg/m^3^ increase in CAP concentration during a resting period 20 hr postexposure (*p* = 0.05) (Figure 6). We observed no statistically significant changes in any of the other HRV end points at either postexposure measurement and no changes in the 24-hr end points measurements.

## Discussion

The recent development of second-generation particle concentrators has made it possible to examine the effects of real-time exposure to atmospheres in which size-fractionated PM is selectively concentrated. This study shows that an acute exposure of healthy young adults to concentrated coarse CAPs resulted in significant changes in indices of pulmonary inflammation, hemostasis, and autonomic nervous system balance. The mean coarse PM concentration (105.1 μg/m^3^) measured at the inlet to the exposure chamber in this study is not unrealistically high and can be found in many areas throughout the world, including locations in the U.S. Southwest. Furthermore, the actual PM concentration at the mouth of the subject was only 67% of that measured at the chamber inlet, resulting in average exposures to only 70.3 μg/m^3^. Table 1 shows the actual dose of PM inhaled by each person, taking ventilation into account.

Coarse PM are preferentially deposited in the proximal region of the lung. They also contain the bulk of particle-bound biological material such as lipopolysaccharide, which is a known proinflammatory agent. Therefore, we expected that coarse CAP exposure would elicit an inflammatory response (PMN influx) in the lung. Indeed, we did find a small but statistically significant increase in the percentage of PMNs in the BAL fluid. In a previous study, we exposed young, healthy volunteers to fine CAPs (PM_2.5_), divided into four concentrations quartiles of 0 (filtered air), 47.2, 107.4, and 206.7 μg/m^3^ (Ghio et al. 2000). In the present study, the mean PM concentration (105.1 μg/m^3^) was very close to the mean concentration of the third quartile in the fine CAP study (107.4 μg/m^3^) and resulted in an identical percentage of PMNs in the BAL fluid, suggesting that coarse and fine PM may be equally potent in inducing pulmonary inflammation. Other studies have not reported an influx of inflammatory cells into the lung after exposure of humans to CAPs. However, those studies used induced sputum to obtain cells from the respiratory tract rather than BAL, and we have found the latter is a more sensitive and less noisy method to measure lung inflammation, particularly when the percent influx of PMNs is small.

We did not observe decreases in lung function in this study, nor did we observe increases in soluble markers of pulmonary inflammation such as IL-6, IL-8, or PGE_2_. This is in agreement with other studies in which both healthy humans and those with pulmonary disease have been exposed to CAPs (Ghio et al. 2000; Gong et al. 2003a, 2003b, 2004a, 2008) and strengthens the notion that acute exposure to air pollution particles generally does not seem to result in substantial changes to the respiratory system. In contrast to CAP exposure, our previous studies in which humans were exposed to low levels of ozone have shown a marked decrease in pulmonary function and increase in inflammatory cells and cytokines (Devlin et al. 1991; Horstman et al. 1990), suggesting that particles and ozone exert their effects via dissimilar mechanisms.

In this study, we found decreased blood plasma tPA levels 20 hr after exposure to coarse CAPs. tPA is a protein that is involved in the breakdown of blood clots by catalyzing the conversion of plasminogen to plasmin, the major enzyme responsible for clot breakdown. Decreased tPA levels could inhibit the breakdown of any clots formed by particles, thus increasing the odds of a thromboembolic event. These findings add to the growing number of studies that have found associations between PM exposure and alterations in indices of hemostasis and thrombosis. We previously reported elevations in blood fibrinogen, a fibrin precursor and acute phase reactant, in the blood of healthy volunteers 24 hr after exposure to fine CAPs (Ghio et al. 2003). Increased blood fibrinogen has also been associated with exposure to air pollution in a number of panel studies (Chuang et al. 2007; Liao et al. 2005; Pekkanen et al. 2000; Ruckerl et al. 2007). We also reported an association between PM and vWF, a glycoprotein involved in endothelial cell activation, hemostasis, and platelet adhesion, in a panel of highway patrol troopers exposed to near-road air pollution (Riediker et al. 2004). Others have also reported associations between PM exposure and increased vWF (Liao et al. 2005), as well as plasminogen activator inhibitor (Chuang et al. 2007; Mills et al. 2005; Su et al. 2006). Taken as a whole, these studies indicate that exposure to PM has the potential to alter hemostatic balance function in the blood, favoring a prothrombogenic environment and interfering with fibrinolytic pathways. Whether these changes in hemostatic factors contribute to the triggering of cardiovascular and other thrombotic events after PM exposure remains to be established.

Analysis of HRV is a noninvasive method to assess the function of the autonomic nervous system. Reduced HRV is considered a prognostic marker for adverse cardiovascular events in patients with a prior myocardial infarction. Panel studies have consistently associated fine PM exposure with decreased HRV. Gold et al. (2000) reported an association between decreased SDNN and increased PM_2.5_, as did Liao et al. (1999) and Pope et al. (1999). Previous work from our group demonstrated that healthy elderly individuals without overt cardiovascular disease exposed to fine CAPs experienced decreased SDNN and HF HRV in 5-min resting intervals immediately and 24 hr after exposure (Devlin et al. 2003). A similar study by Gong et al. (2004a) involving healthy elderly individuals exposed to fine CAPs reported a significant decrease in SDNN 18 hr after exposure.

In this study we extend our earlier findings by showing that exposure to coarse CAPs also results in decreased SDNN 20 hr after exposure. These results are in agreement with an earlier study which also reported decreased SDNN in healthy volunteers exposed to coarse CAPs (Gong et al. 2004b). They are also in agreement with a recent panel study in which we reported an association between coarse PM and decreased SDNN in asthmatics (Yeatts et al. 2007).

A note of caution must be exercised in interpreting these findings. We measured multiple end points in the lung, blood, and heart. Therefore, some of the statistically significant findings may have been due to chance alone. It will be important to see whether these findings can be replicated in our own and others’ studies.

There has been considerable discussion about the pathways by which PM can cause acute cardiovascular changes. One of the early thoughts was that particles could cause changes to the primary target organ, the lung, which would spill over into the vascular system and secondarily affect autonomic function. However, data from numerous human and animal toxicology studies suggest that CAPs do not induce sufficient pulmonary responses to cause these kinds of secondary effects. Indeed, if this were the case, one might expect that ozone, a powerful inducer of pulmonary inflammation, would be able to cause even more substantial cardiovascular changes than PM, which has not been shown to date.

Recent studies in rodents have hypothesized that ultrafine PM (PM_0.1_; those < 0.1 μm in diameter) may actually leave the lung and directly attack the cardiovascular system (Nemmar et al. 2002; Oberdorster et al. 2002). Although this mechanism may play a role in cardiovascular effects caused by ultrafine PM, the large size of coarse PM makes it unlikely that they can pass directly into the circulation. However, it does not exclude the possibility that soluble components of coarse PM may find their way into the circulatory system. Recent *in vitro* experiments in our laboratory have shown that 40% of the activity of coarse PM that affects cultured airway epithelial cells resides in the water-soluble portion of the PM (data not shown).

A third possibility is that PM, regardless of size, may affect the cardiovascular system through nerve impulses transmitted from the lung to the brain. Ozone-induced decrements in lung function are thought to be mediated via interaction of the pollutant with C-fibers innervating the lung in humans and dogs (Bromberg and Koren 1995; Coleridge et al. 1993; Passannante et al. 1998). Administration of β-adrenergic receptor and muscarinic receptor antagonists effectively blocked PM-induced cardiac oxidative stress in rats (Rhoden et al. 2005). Capsazepine, a selective antagonist of the vanilloid receptor present on pulmonary C-fibers, blunt PM-induced changes in cardiac oxidative stress and edema in rats (Ghelfi et al. 2008). Tunnicliffe et al. (2001) reported HRV changes but no respiratory system changes in humans exposed to sulfur dioxide, suggesting that a cardiac autonomic effect can be triggered by upper-airway irritant receptors.

## Conclusions

The results of this study showed that young, healthy people experience mild acute physiologic effects when exposed to environmentally relevant coarse air pollution PM. The results of this study are generally consistent with those of previously published studies examining the effects of both coarse and fine PM, suggesting that both particle size fractions are roughly equivalent in inducing cardiopulmonary changes in healthy humans. However, given the large number of end points typically measured in these studies, the relatively small number of positive findings makes it important for these findings to be replicated in future studies.

## Figures and Tables

**Table 1:** Basic demographic and PM concentration data.

				PM concentration (μg/m^3^)		Calculated PM dose (μg)^a^	
Subject	Sex	Age (years)	Minute ventilation (L/min)	Total	Coarse	Total	Coarse
1	Male	29	21.7	170.3	159.6	297	278
2	Male	21	23.1	73.4	60.3	136	112
3	Female	22	20.1	232.8	196.9	375	317
4	Male	23	18.4	168.9	136.9	250	203
5	Male	26	21.3	100.0	75.0	171	128
6	Female	23	12.2	106.3	85.8	104	84
7	Female	22	26.1	56.1	56.1	117	117
8	Male	34	17.0	127.3	102.8	174	141
9	Male	28	26.2	27.4	23.7	58	50
10	Female	22	26.0	115.4	85.4	241	178
11	Male	34	22.7	120.9	106.2	220	193
12	Female	24	27.4	98.0	88.8	216	196
13	Female	20	24.5	36.5	31.8	63	55
14	Male	20	17.4	38.3	36.1	54	51
Mean ± SD		24.9 ± 4.7	21.5 ± 4.3	105.1 ± 58.3	89.0 ± 49.5	176.7 ± 95.9	150.3 ± 82.5

a Calculated PM dose (μg) = (minute ventilation × 120 min)/1,000 L × PM concentration × 0.67 (the estimated proportion of the particles being delivered to the airway, calculated by comparing PM values at the inlet to the chamber and a few inches away from a subject’s mouth).
